# Psychometric validation of the Worst Itch Numerical Rating Scale (WI-NRS) and other patient-reported outcome measures for assessing severity and impact of pruritus in patients with primary biliary cholangitis

**DOI:** 10.1186/s13023-025-03798-x

**Published:** 2025-07-31

**Authors:** Heather Gelhorn, Brooke M. Currie, Donald M. Bushnell, Mona L. Martin, Fatoumata Fofana, Hayley Karn, Marlyn J. Mayo, David E. Jones, Megan M. McLaughlin, Robyn von Maltzahn

**Affiliations:** 1 Evidera|PPD, Wilmington, NC USA; 2 GSK, Collegeville, PA USA; 3 Evidera|PPD, Paris, France; 4 Evidera|PPD, London, UK; 5 University of Texas Southwestern Medical Center, Dallas, TX USA; 6 Newcastle University, Newcastle, UK; 7 GSK, London, UK

**Keywords:** 5-D itch scale, Itch, Patient-reported outcomes, PBC-40, Primary biliary cholangitis, Cholestatic pruritus, Psychometric validation, Numerical rating scale, Fatigue, Sleep interference

## Abstract

**Background:**

Cholestatic pruritus is common in primary biliary cholangitis (PBC), often leading to sleep disturbances and substantially impairing health-related quality of life (HRQoL). Fatigue, also frequent in PBC, can be exacerbated by sleep interference due to nighttime pruritus. Quantitative numerical rating scales (NRS) are appropriate for assessing unidimensional patient-reported outcome (PRO) concepts and are easily interpreted. The PBC-40 is a disease-specific, patient-derived 40-item tool that assesses HRQoL in patients with PBC. To assess pruritus severity in clinical trials, rigorous validation of PROs in the target patient population is required. This study aimed to validate the NRS items for worst itch (WI-NRS), pruritus-related sleep interference (Sleep Interference NRS), and fatigue (Fatigue NRS), plus the PBC-40 (modified with a 7-day recall), among patients with PBC and pruritus.

**Methodology:**

Data were analyzed from the Phase 2b GLIMMER trial (NCT02966834) of linerixibat and a separate observational study in patients with PBC and pruritus. Pruritus severity was assessed using the 0–10 WI-NRS. Psychometric properties of the WI-NRS, Sleep Interference NRS, Fatigue NRS, and PBC-40 (7-day recall) were evaluated. Additional PRO data were used to support validation analyses. Multiple psychometric methods were used to validate the NRS items and PBC-40 (7-day recall).

**Results:**

Data for 288 patients (n=147 GLIMMER; n=141 observational study) with PBC experiencing pruritus were included. The internal consistency of PBC-40 (7-day recall) was acceptable to excellent and confirmatory factor analysis confirmed its domain structure. All PBC-40 domains showed acceptable test–retest reliability (intraclass correlation coefficients [ICCs]: 0.72–0.90). The WI-NRS showed acceptable test–retest reliability (ICCs: 0.73–0.81 [GLIMMER]; ICC: 0.78 [observational study]). Test–retest reliability was also acceptable for the Sleep Interference (ICC: 0.85 [GLIMMER]; ICC: 0.77 [observational study]) and Fatigue (ICC: 0.88 [GLIMMER]; ICC: 0.78 [observational study]) NRS items. Convergent, discriminant, and known-groups validity were confirmed for all three NRS items and the PBC-40. Additionally, the NRS and PBC-40 Itch domain demonstrated responsiveness by reflecting change in other PROs over time.

**Conclusions:**

The data support the psychometric reliability, validity, and responsiveness of WI-NRS, pruritus-related Sleep Interference NRS, and Fatigue NRS, and PBC-40 (7-day recall) in patients with PBC experiencing pruritus.

**Supplementary Information:**

The online version contains supplementary material available at 10.1186/s13023-025-03798-x.

## Plain English summary 

Primary biliary cholangitis (PBC) is a rare liver disease in which flow of bile (a fluid from the liver involved in digestion) is disturbed. Itching (also known as pruritus), sleep disturbance, and fatigue are common problems of PBC that can dramatically reduce a person’s quality of life. Questionnaires and numbered scales, known as numerical rating scales, can be used to measure symptoms of PBC. To ensure they accurately capture symptom severity and impacts on quality of life, they need to be thoroughly tested in the group of people they will be used in. This process is known as psychometric validation.

This study aimed to validate, in people with PBC, three numerical rating scale questions (ranging from 0 to 10) measuring itch, sleep interference related to itch, and fatigue, plus a modified version of a questionnaire known as the PBC-40 (designed to measure quality of life in people with PBC). Results for these measures from two studies (one clinical and one survey) were analyzed and compared with the findings from other questionnaires used in these studies.

The authors concluded that the three numerical rating scale questions and the modified PBC-40 questionnaire accurately measure symptom severity, including itch and its impact on sleep, fatigue, and quality of life in people with PBC and pruritus. This supports the use of these measures in clinical trials for PBC, and if used regularly in clinical practice, may facilitate the management of itch in people with PBC by enhancing communication between patients and medical care providers.

## Background

Primary biliary cholangitis (PBC) is a rare, chronic, autoimmune cholestatic liver disease [1]. Cholestatic pruritus (itch) is common in PBC, affecting up to 89% of patients at some point during the course of their disease [2–6]. Pruritus substantially impairs health-related quality of life (HRQoL) in patients with PBC, and often leads to sleep disturbance [2, 4, 7]. However, despite its frequency and impact, pruritus is often not documented in medical records [6]. Fatigue is also common in PBC [8]. This symptom can be exacerbated by many factors, including sleep interference due to nighttime pruritus [3, 6, 9].

A variety of response scales have been used in patient-reported outcome (PRO) measures, such as the Likert response scale, numerical rating scale (NRS), verbal rating scale (VRS), and visual analog scale (VAS) [10]. Some response scales may be more suitable than others depending on the question being asked and the planned analysis and use of the data. The NRS, VAS, and VRS are response scales that have been used historically to assess pruritus severity in a variety of patient groups, including those with advanced chronic kidney disease [11], atopic dermatitis [12, 13], and prurigo nodularis [14]. The 0–10 NRS is an especially appropriate scale for assessing unidimensional symptom concepts such as pain or pruritus, as it is widely used in clinical practice and research and is easily interpreted by respondents [15]. Furthermore, the US Food and Drug Administration (FDA) is often amenable to the use of NRS as endpoint measures to support labeling claims [16–18]. The Worst Itch NRS (WI-NRS) is a single-item PRO measure in which patients indicate the intensity of the worst itching they experienced over a specified period of time on a scale of 0 (no itching) to 10 (worst imaginable itching) [7, 19]. This NRS has been used in clinical trials to assess itch in PBC, as well as in other cholestatic liver conditions associated with pruritus [19–27]. The WI-NRS aligns well with the Brief Pain Inventory (BPI). The BPI is a PRO instrument that has been widely and successfully used as a measure of pain intensity in clinical trials and uses a 0–10 NRS to assess average pain as well as worst pain [28].

The PBC-40 is a disease-specific tool designed to assess HRQoL in patients with PBC [29–31] and is extensively used in clinical [7, 32] and nonclinical research in PBC [6, 33]. It comprises 40 question items grouped into six distinct PBC-specific health domains of Symptoms, Itch, Fatigue, and Cognitive, Social, and Emotional functioning [29–31]. This questionnaire was originally validated using a 4-week recall period where it showed good psychometric properties, and a high level of relevance to PBC [29, 30]. The tool has been developed and validated in patients both with and without pruritus [29, 30]. Qualitative assessment of an adapted PBC-40 instrument with a 7-day recall has previously been conducted in patients with itching associated with PBC as part of an initial validation step [30], though additional quantitative and psychometric assessments of the instrument are still necessary for full validation.

Based on current peer-reviewed literature and to the best of our knowledge, there is limited research published to date that validates PRO instruments in patients with cholestatic pruritus and PBC. Rigorous development and psychometric validation of PRO instruments in the target patient population is essential, particularly to confirm their ability to quantify change in the context of therapeutic intervention. The objective of this study was to conduct a psychometric validation of several PRO measures. These included three NRS items: Worst Itch, pruritus-related Sleep Interference, and Fatigue (hereafter referred to as WI-NRS, Sleep Interference NRS, and Fatigue NRS) and the PBC-40 modified with a 7-day recall (hereafter referred to as PBC-40 [7-day recall]).

## Methods

### Study design and patient population

This analysis was based on data from the Phase 2b GLIMMER trial (NCT02966834; GSK study 201000), and a separate observational study (GSK study 212144) in patients with PBC.

Details of the GLIMMER study design have been published elsewhere [19]. The study ran from January 11, 2017, to April 15, 2020. In brief, GLIMMER was a global, randomized, placebo-controlled study of linerixibat in patients aged 18–80 years with clinically confirmed PBC and moderate-to-severe pruritus. The study comprised screening, a 4-week placebo run-in, a 12-week double-blind treatment period (baseline/Week 4 to Week 16), followed by a 4-week single-blind placebo period (Week 16 to Week 20) and follow-up period (Week 20 to Week 24). Participants were eligible if they had rated their pruritus severity as ≥ 4 on a 0–10 itch NRS for the majority of the time during the 8 weeks prior to screening. Following the placebo run-in, the protocol permitted patients with a WI-NRS score (weekly itch score) ≥ 3 to be randomized to either linerixibat or placebo at baseline (Week 4). Consequently, 24% of the population entered the 12-week treatment period with pruritus severity close to, but not quite moderate (WI-NRS ≥ 3 to < 4) [19].

For the observational study, data were collected from a sample of patients who self-reported a clinical diagnosis of PBC, both with and without current pruritus. Recruitment was completed with the support of a market research vendor who had access to a large online community of patients with PBC identified for research panels in the United States, the United Kingdom, and Canada. While all patients had previously experienced pruritus, efforts were made to ensure approximately 70% of the sample currently had pruritus. Participants completed a daily web-survey over 8 consecutive days.

### Assessments and analyses

Psychometric properties of the WI-NRS, Sleep Interference NRS, Fatigue NRS, and the PBC-40 (7-day recall) were assessed using data pooled across treatment groups from the GLIMMER study and supporting data from the observational study. In GLIMMER, all analyses were conducted on the intent-to-treat population who had available PRO assessments at baseline; both treatment (all doses) and placebo arms were pooled for the analyses.

Symptoms were recorded by participants in an eDiary questionnaire twice daily (morning and evening) from Day 1 to Week 20 in the GLIMMER study and from Day 1 to Day 8 in the observational study. In both studies, participants recorded the severity of their worst itch using a 0–10 NRS, where 0 = no itching and 10 = worst imaginable itching. The worst of these two daily scores was documented as the worst daily itch score. The weekly itch score (previously referred to as mean worst daily itch [19]) was calculated from the average worst daily itch scores over a 7-day period. Two additional 0–10 NRS items were used once daily to document pruritus-related Sleep Interference (0 = did not interfere; 10 = completely interfered) and Fatigue (0 = no fatigue; 10 = worst possible fatigue). The Sleep Interference NRS was assessed in the morning (with patients reflecting on their sleep from the night before), while the Fatigue NRS was assessed in the evening (with patients reflecting on their fatigue for that day). Weekly sleep and fatigue scores were calculated as the average of daily scores over a 7-day period.

In GLIMMER, the PBC-40 (7-day recall) was administered monthly at each study visit, from Visit 2 (Day 1) to Visit 7 (Week 20), for the entire study period; in the observational study, participants completed the PBC-40 (7-day recall) at Days 1 and 8.

Additional PROs, including the 5-dimension (5-D) itch scale [34], EQ-5D [35], Patient Global Impression of Severity (PGI-S), and Patient Global Impression of Change (PGI-C)—the latter two being specific to itch and developed specifically for the linerixibat program—were used to support validation analyses. PGI-C included a follow-up question on meaningfulness of change where participants reported either Yes or No to whether they found their change in itch to be meaningful. A brief description of each of these instruments and schedule of their use is described in Supplementary Tables 1 and 2.

### Psychometric analyses

Validation of the NRS items and PBC-40 (7-day recall) was carried out using a variety of psychometric methods. First, internal consistency reliability of the PBC-40 (7-day recall) was examined to understand the extent to which individual items within the measure were related to each other and with the scale as a whole. This measure of reliability was examined using Cronbach’s coefficient alpha, with values > 0.70 generally considered acceptable for aggregate data [36]. Second, confirmatory factor analysis of data from the observational study evaluated dimensionality and existing structure of the PBC-40 (7-day recall) using Day 1 data to test the Fatigue, Emotional, Social, Cognitive, Symptoms, and Itch domains. Fit statistics (root mean square error of approximation [RMSEA], and comparative fit index [CFI]) were examined to assess model goodness-of-fit. RMSEA values < 0.08 and CFI > 0.90 denoted good model fit [37–39]. Third, test–retest reliability of the NRS items and PBC-40 (7-day recall), was evaluated to examine their stability and reproducibility between two time points within a stable population. Participants who selected the same PGI-S response between the test and retest time points were defined as stable and included in the analysis. This involved using paired t-tests and intraclass correlation coefficients (ICC), where ICC values > 0.70 are generally considered acceptable for establishing test−retest reliability. Additionally, construct validity (including convergent, discriminant, and known-groups validity) was assessed against other PRO measures. Convergent validity included demonstrating that different measures of the same or similar concepts correlated with one another. Divergent validity analysis confirmed that measures or items assessing dissimilar concepts did not substantially correlate with one another. Spearman rank-order correlations were used for convergent/discriminant validity. The strength of the correlations for convergent/divergent validity analyses were described as small if < 0.3, moderate if 0.3–0.6, and large if > 0.6 for both positive and negative correlations. Known-groups validity measured the extent to which scores from an instrument were distinguishable between groups of subjects who differed by a relevant clinical indicator or other parameter. Analysis of variance (ANOVA) (adjusted for multiple comparisons based on Tukey method) was used for known-groups validity, with the F-statistic considered significant below a critical value of 0.05. Finally, the ability of the PROs to detect change over time (i.e. their responsiveness) was analysed by reviewing the association between change from baseline to Week 16 in the NRS items and PBC-40 (7-day recall) with PGI-C categories and change from baseline in PGI-S, using data from GLIMMER. Analysis of covariance (ANCOVA) and paired t-tests were used to test differences in mean scores and compare effect size statistics.

## Results

### Sample description

Patient demographics and clinical characteristics for both studies are presented in Table 1. Patients in GLIMMER (*N* = 147) had a mean (standard deviation [SD]) age of 55.8 (11.0) years, 70% were white, and 94% were female. Mean (SD) duration of PBC and pruritus was 8.1 (6.7) years and 8.1 (7.9) years, respectively [19]. At baseline, the mean (SD) WI-NRS (weekly itch score; scale 0–10) was 5.5 (1.8). In the observational study (*N* = 141), the majority of participants were older than 35 years (*n* = 131, 93%), white (87%), and female (93%). All participants had PBC for longer than 1 year, with most of them experiencing pruritus for more than 1 year (*n* = 112, 79%), and 19 (13%) of them having pruritus for more than 10 years. At the time of enrollment (screening), 69% were currently experiencing pruritus with a mean (SD) WI-NRS (scale 0–10) of 4.7 (2.8). Furthermore, participants in the observational study were asked to rate the severity of their itch at the start of the study (Day 1). Participants rated their overall pruritus severity as very mild/mild (47%), moderate-to-severe (50%), and 3% reported having none.

**Table 1:** Patient demographics and clinical characteristics for both GLIMMER^a^ and observational study^b^

	GLIMMER (*N* = 147)	Observational study (*N* = 141)
Female, *n* (%)	138 (94)	131 (93)
Age, mean (SD), years	55.8 (11.0)	-
Older than aged 35 years, n (%)	-	131 (93)
Race, n (%)		
White	103 (70)	122 (87)
Asian	38 (26)	4 (2.8)
Japanese	38 (26)	
Other/not captured	6 (4)	15 (11)
ALP x ULN		
Mean (SD)	1.6 (1.4)	-
Median (IQR)	1.1 (0.4, 11.5)	-
Duration of PBC, mean (SD), years	8.1 (6.7)	-
Diagnosis of PBC ≥ 12 months, n (%)		141 (100)
Duration of pruritus, mean (SD), years^c^	8.1 (7.9)	-
Experienced pruritus for over 1 year, n (%)		112 (79)
Experienced pruritus for over 10 years, n (%)		19 (13)
Severity of pruritus at baseline, n (%)		
Mild (< 4)^d^	35 (24)	-
Moderate (≥ 4–< 7)	76 (52)	-
Severe (≥ 7–10)	36 (24)	-
Severity of pruritus during the past week, mean (SD)^e^		4.4 (2.7)
Severity of pruritus on Day 1, n (%)		
Very mild	-	20 (14)
Mild	-	46 (33)
Moderate	-	56 (40)
Severe	-	11 (8)
Very severe	-	4 (3)
None	-	4 (3)
WI-NRS at baseline, mean (SD)^f^	5.5 (1.8)	-
WI-NRS at screening, mean (SD)		4.7 (2.8)
Receiving UDCA, n (%)	137 (93)	137 (97)
Receiving obeticholic acid, n (%)	-	17 (12)
Receiving other treatments that may impact pruritus		
Rifampin	2 (1)	7 (5)
Fibrates (bezafibrate, fenofibrate)	3 (2)	7 (5)
Sertraline	4 (3)	6 (4)
Gabapentin	1 (5)	6 (4)
Cholestyramine or other resin	-	5 (4)

^a^Safety population, Week 4 (baseline) [19]

^b^All patients, Day 1

^c^Pruritus duration was determined from date of onset of pruritus to date of screening

^d^Patients with NRS ≥ 4 most of the time during the 8 weeks before screening were included in the study; however, short periods of NRS < 4 were acceptable, so long as the worst daily itch score was ≥ 4 for at least half the days, as recalled by the participant

^e^On a scale from 0 to 10 where 0 = no itching at all to 10 = worst imaginable itching

^f^Weekly itch score: the average of worst daily itch scores over a 7-day period, previously referred to as mean worst daily itch score [19]

ALP, Alkaline phosphatase; IQR, Interquartile range; NRS, Numerical rating scale; PBC, Primary biliary cholangitis; SD, Standard deviation; UDCA, Ursodeoxycholic acid; ULN, Upper limit of normal; WI-NRS, Worst Itch NRS

### Confirmatory factor analysis

In the observational study, confirmatory factor analysis confirmed the PBC-40 (7-day recall) domain structure with factor coefficients all being ≥ 0.39 and adequate fit statistics (RMSEA = 0.059, CFI = 0.966) (Supplementary Table 3).

### Internal consistency reliability

In GLIMMER, the internal consistency reliability of PBC-40 (7-day recall) was acceptable to excellent (Cronbach’s alpha 0.76–0.97) at baseline except for the Symptoms domain which had a slightly lower Cronbach’s alpha (0.67) than other domains (Supplementary Table 4). Similarly, in the observational study, internal consistency reliability of PBC-40 (7-day recall) was excellent (overall Cronbach’s alpha = 0.95) and was acceptable to excellent for all six domains at Day 1 (Cronbach’s alpha 0.73–0.95) (Supplementary Table 4).

### Test–retest reliability

The WI-NRS showed acceptable test–retest reliability in GLIMMER between Days 2–8 and Days 9–15, and between Day 2 and baseline/Week 4 in stable participants (ICCs: 0.73–0.81) and in the observational study between Day 1 and Day 8 in a sample of stable participants (ICC: 0.78) (Supplementary Tables 5 and 6). The test–retest reliability was also acceptable for the Sleep Interference (ICC: 0.85) and Fatigue (ICC: 0.88) NRS items between Day 2 and 1 week later in GLIMMER (Supplementary Table 5), and similarly in the observational study between Day 1 and Day 8 in a sample of stable participants (Sleep Interference NRS, ICC: 0.77; Fatigue NRS, ICC: 0.78; Supplementary Table 6). All PBC-40 (7-day recall) domains showed acceptable test–retest reliability between Days −21 to −7 and Day 1 in stable participants in GLIMMER (ICCs: 0.72–0.87) and in the observational study between Day 1 and Day 8 in a sample of stable participants (ICCs: 0.85–0.90) (Supplementary Table 7).

### Construct validity

#### Convergent/discriminant validity

In GLIMMER, convergent validity of all three NRS items was confirmed based on moderate–large correlations with scores of similar concepts from other measures. For example, correlations were observed between WI-NRS weekly itch score and 5-D Itch scale Degree item score, PBC-40 (7-day recall) Itch domain score and 5-D Itch scale total score, Sleep Interference NRS and 5-D Itch scale Sleep item score, and Fatigue NRS and PBC-40 (7-day recall) Fatigue domain score and BDI-II loss of energy and tiredness/fatigue item scores (*r* = 0.46–0.69; all *p* < 0.0001) (Table 2). The WI-NRS showed good discriminant validity based on low to slightly moderate correlations (*r* = |0.21| to |0.37|) with scores of dissimilar measures or concepts such as BDI-II total score, PBC-40 (7-day recall) Emotional, Social, Cognitive, or Symptoms domain scores and EQ-5D utility and VAS scores (Table 3).

**Table 2:** Convergent validity of the NRS items at baseline in GLIMMER and the observational study

	*r* ^a^
**WI-NRS (GLIMMER)** ^**b**^	
5-D Itch scale Degree score	0.61****
PBC-40 Itch domain score	0.50****
5-D Itch scale total score	0.59****
**WI-NRS (observational study)** ^**c**^	
5-D Itch scale total score	0.71****
PBC-40 Itch domain score	0.79****
**Sleep Interference NRS (GLIMMER)**	
5-D Itch scale Sleep item score	0.58****
**Sleep Interference NRS (observational study)** ^**d**^	
PBC-40 Sleep item 8 score	0.79****
**Fatigue NRS (GLIMMER)**	
PBC-40 Fatigue domain score	0.69****
BDI-II loss of energy item score	0.46****
BDI-II tiredness or fatigue item score	0.51****
**Fatigue NRS (observational study)** ^**e**^	
PBC-40 Fatigue domain score	0.68****

^a^Significance levels of Spearman rank-order correlations, all ****p < 0.0001

^b^Weekly itch score

^c^Worst daily itch score

^d^Daily sleep score

^e^Daily fatigue score

5-D, 5-dimension; BDI-II, Beck Depression Inventory - II; NRS, Numerical rating scale; PBC-40, Primary Biliary Cholangitis – 40 items; WI-NRS, Worst Itch NRS

**Table 3:** Discriminant validity of WI-NRS at baseline in GLIMMER^a^

Domain score / Score description	*N*	*r* ^b^
BDI-II total score	147	0.26**
PBC-40 Emotional domain score	147	0.22**
PBC-40 Social domain score	147	0.30***
PBC-40 Cognitive domain score	147	0.32****
PBC-40 Symptoms domain score	147	0.31***
EQ-5D Utility score	146	−0.37****
EQ-5D VAS score	146	−0.21*

^a^Weekly itch score

^b^Significance levels of Spearman rank-order correlations, *p*-values are: *p < 0.05, **p < 0.01, ***p < 0.001, ****p < 0.0001

5-D, 5-dimension; BDI-II, Beck Depression Inventory - II; NRS, Numerical rating scale; PBC-40, Primary Biliary Cholangitis – 40 items; VAS, Visual analog scale; WI-NRS, Worst Itch NRS

Convergent validity of all three NRS items in the observational study was evidenced by large, positive correlations with scores of similar concepts from other measures (*r* ≥ 0.68, all *p* < 0.0001) (Table 2). The WI-NRS daily itch score showed convergent validity with the 5-D Itch scale total score and PBC-40 (7-day recall) Itch domain score, and Sleep Interference NRS with PBC-40 (7-day recall) Sleep item 8 score, and Fatigue NRS with PBC-40 (7-day recall) Fatigue domain score. Convergent validity of PBC-40 (7-day recall) was demonstrated across similar measures for the Itch, Fatigue, Cognitive, and Emotional domains based on large correlations with scores of similar concepts from other measures; for example, 5-D Itch scale total score and WI-NRS weekly itch score in relation to the PBC-40 Itch domain (all *r* ≥ 0.50 with *p* < 0.0001) in GLIMMER (Supplementary Table 8). Similarly, in the observational study, convergent validity of PBC-40 (7-day recall) was supported for the Itch, Fatigue, Cognitive, Social, and Emotional domains based on large correlations with scores of similar concepts from other measures (all *r* ≥ 0.61 with *p* < 0.0001). The only exception was a smaller but significant correlation between the PBC-40 (7-day recall) Fatigue domain and the Sleep Interference NRS item (*r* = 0.27, *p* < 0.01) (Supplementary Table 8).

#### Known-groups validity

**Fig. 1:**
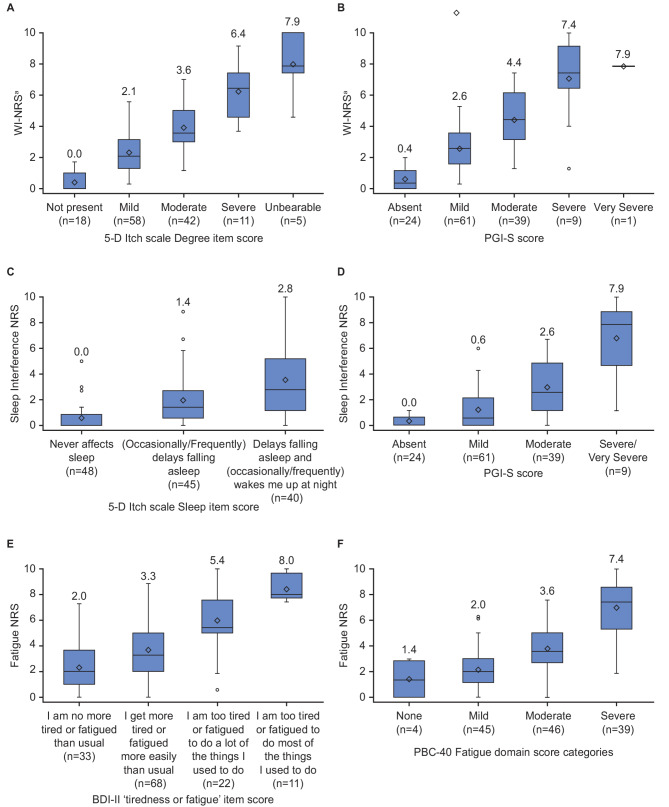
Known-groups validity boxplots of key NRS items at week 16 in GLIMMER versus other measures. WI-NRS weekly itch score by (**A**) 5-D itch scale Degree item and (**B**) PGI-S. Sleep Interference NRS by (**C**) 5-D itch scale Sleep item and (**D**) PGI-S. Fatigue NRS by (**E**) BDI-II tiredness or fatigue item and (**F**) PBC-40 Fatigue domain. The numbers above the box plots represent medians. ^a^Weekly itch score. 5-D, 5-dimension; BDI-II, Beck Depression Inventory – II; NRS, Numerical rating scale; PBC-40, Primary Biliary Cholangitis – 40 items; PGI-S, Patient Global Impression of Severity; WI-NRS, Worst Itch Numerical Rating Scale

Known-groups validity was confirmed in GLIMMER; all NRS items discriminated between levels of severity in the expected direction across relevant measures including known groups based on the PGI-S, 5-D Itch scale Degree item (for WI-NRS weekly itch score), 5-D Itch scale Sleep item (for Sleep Interference NRS), and BDI-II tiredness/fatigue item and PBC-40 Fatigue domain (for Fatigue NRS) (Fig. 1). These overall comparisons were significant (all *p* < 0.0001). Similarly, in the observational study, known-groups validity of the NRS items was confirmed based on groups defined by the PGI-S (all *p* < 0.001) (Supplementary Table 9).

Known-groups validity for the PBC-40 (7-day recall) was confirmed. At Week 16 in GLIMMER, all PBC-40 (7-day recall) domains discriminated well between patients in the expected direction according to groups defined by an item related to tiredness interference with daily activities and the EQ-5D VAS (all *p* < 0.01) (Supplementary Fig. 1). Trends in means for each group were consistently in the expected direction.

Known-groups validity for PBC-40 (7-day recall) was also confirmed in the observational study. At Day 1, all PBC-40 (7-day recall) domains discriminated between patients in the expected direction according to groups defined by PGI-S with the Itch, Fatigue, Social, and Emotional domains being highly discriminant (*p* < 0.001). When examining groups by the General Health item of the PBC-40 (7-day recall), five domains (Fatigue, Emotional, Social, Cognitive, and Symptoms) were able to differentiate between levels of health (*p* < 0.001).

### Responsiveness

NRS items in GLIMMER demonstrated an ability to detect change over time by reflecting changes in other PRO measures in the expected direction (Table 4). Among patients who indicated the changes on the PGI-C were meaningful to them, both the WI-NRS (*p* < 0.0001) and the Sleep Interference NRS (*p* < 0.0001) showed significant differences in the mean changes by PGI-C response group, in the anticipated direction. Furthermore, responsiveness of the Fatigue NRS was confirmed through examining the association between change from baseline to Week 16 in the weekly Fatigue NRS score and different levels of change in the PBC-40 Fatigue domain score (*p* < 0.0001). Similarly, the PBC-40 (7-day recall) Itch domain was supported by all levels of change from baseline in the PGI-C and PGI-S in the expected direction (both *p* < 0.0001) in GLIMMER (Table 4 and Supplementary Table 10).

**Table 4:** Responsiveness of the NRS items and the PBC-40 Itch domain in GLIMMER

	*N*	Baseline mean (SD)	Week 16, mean (SD)	Change, mean (SD) (Week 16 - baseline)	Effect Size^a^	SRM^b^	Paired t-test (*p*-value)^c^	F Value (*p*-value)^d^
WI-NRS								
**PGI-C + meaningful question (Yes/No)** ^**e, f**^								19.93 (< 0.0001)
Very much improved + Yes	55	5.4 (1.7)	1.6 (1.6)	−3.8 (2.1)	−2.2	−1.8	−13.6 (< 0.0001)	
Moderately improved + Yes	30	5.7 (1.8)	3.6 (1.5)	−2.2 (1.9)	−1.2	−1.2	−6.36 (< 0.0001)	
Minimally improved + Yes	14	5.5 (2.1)	4.9 (1.9)	−0.6 (2.3)	−0.3	−0.3	−0.98 (0.3429)	

**Sleep Interference NRS**								
**PGI-C + meaningful question (Yes/No)** ^**e**^								16.68 (< 0.0001)
Very much improved + Yes	55	3.6 (2.3)	0.8 (1.5)	−2.8 (2.0)	−1.3	−1.4	−10.4 (< 0.0001)	
Moderately improved + Yes	30	4.6 (2.4)	2.6 (1.9)	−2.0 (2.1)	−0.9	−1.0	−5.34 (< 0.0001)	
Minimally improved + Yes	14	4.5 (3.0)	3.3 (2.4)	−1.2 (1.9)	−0.4	−0.6	−2.37 (0.0337)	

**Fatigue NRS**								
**PBC-40 Fatigue domain – Change category** ^**g**^								8.97 (< 0.0001)
Improved	67	4.7 (1.9)	3.1 (1.9)	−1.6 (1.9)	−0.9	−0.8	−6.86 (< 0.0001)	
Unchanged	15	5.4 (3.2)	4.5 (3.4)	−0.9 (1.4)	−0.3	−0.7	−2.61 (0.0206)	
Worsened	52	5.8 (2.7)	5.3 (2.9)	−0.5 (1.4)	−0.2	−0.3	−2.39 (0.0204)	

**PBC-40 Itch domain score**								
**PGI-C + meaningful question (Yes/No)** ^**e**^								16.26 (< 0.0001)
Very much improved + Yes	56	9.1 (3.1)	4.6 (2.7)	−4.5 (2.9)	−1.5	−1.6	−11.7 (< 0.0001)	
Moderately improved + Yes	30	9.7 (3.0)	7.6 (3.0)	−2.0 (3.1)	−0.7	−0.7	−3.63 (0.0011)	
Minimally improved + Yes	14	9.1 (3.1)	7.4 (2.5)	−1.6 (1.9)	−0.5	−0.8	−3.16 (0.0075)	

Note: For PGI-C (raw categories) + meaningful question (Yes/No): WI-NRS: *n* = 8 patients selected “moderately improved + No” and *n* = 8 patients selected “minimally improved + No;” Sleep Interference NRS: *n* = 8 patients selected “moderately improved + No” and *n* = 8 patients selected “minimally improved + No;” Fatigue NRS: *n* = 8 patients selected “moderately improved + No” and *n* = 8 patients selected “minimally improved + No.” The other groups with *n* = 0 were not included in this table

Meaningful question: participants were asked whether the change reported was meaningful or not (Yes/No)

^a^Mean difference between the baseline and Week 16 score divided by SD of the baseline score

^b^Mean difference between the baseline and Week 16 weekly score divided by SD of the change from baseline score

^c^Paired t-test is for the differences in means between the baseline and the follow-up time point

^d^ANCOVA adjusted for baseline score was used

^e^“No change + Yes” (*n* = 1) was removed from the analysis due to a small sample size

^f^“Very Much Worse” (*n* = 1), “Very Much Worse + Yes” (*n* = 1), “Worsened + Yes” (*n* = 1) were removed from the analysis due to a small sample size

^g^“Improved” (score change < 0), “unchanged” (score change = 0), “worsened” (score change > 0)

ANCOVA, Analysis of covariance; NRS, Numerical rating scale; PBC-40, Primary Biliary Cholangitis 40 items; PGI-C, Patient Global Impression of Change; SD, Standard deviation; SRM, Standardized response mean; WI-NRS, Worst Itch NRS

## Discussion

Using data from the GLIMMER study, a placebo-controlled Phase 2b trial of linerixibat in patients with PBC experiencing moderate-to-severe pruritus, and data from a separate observational study in patients with PBC and a history of pruritus, this analysis psychometrically validated several NRS items relevant to assessing severity and impact of itch in PBC (WI-NRS, Sleep Interference NRS, and Fatigue NRS), as well as the 7-day recall version of the PBC-40 adapted from the original 4-week recall version. This validation has been carried out according to FDA standards and scientific best practices. To our knowledge, this study is the first to fully validate NRS items related to itch severity, pruritus-related sleep interference, and fatigue in a large PBC population with cholestatic pruritus, a symptom that is common in PBC and can substantially impact HRQoL, frequently leading to sleep disturbance and fatigue [2, 7, 9]. The test–retest reliability was acceptable for the Worst Itch, Sleep Interference, and Fatigue NRS items and convergent validity and responsiveness were confirmed for all three NRS items. The known-groups validity analysis showed that all NRS items discriminated against levels of severity in the expected direction across relevant measures. These results expand on previous work establishing the use of itch NRS measures in other pruritic conditions such as atopic dermatitis [40, 41], prurigo nodularis–associated pruritus [42], and chronic kidney disease–associated pruritus [43]. 

All PBC-40 (7-day recall) domains showed acceptable test–retest reliability, and convergent validity was supported for PBC-40 (7-day recall) Itch, Fatigue, Cognitive, Social, and Emotional domains. The known-groups validity analysis showed that all PBC-40 (7-day recall) domains discriminated between levels of severity in the expected direction across relevant measures. The PBC-40 (7-day recall) Itch domain was responsive to changes in other measures over time. Internal consistency reliability was acceptable to excellent for all PBC-40 (7-day recall) domains.

These results support the validity of the WI-NRS, Sleep Interference NRS, and Fatigue NRS items and confirm the reliability and validity of these items as measures of pruritus, sleep interference, and fatigue severity among patients with PBC and pruritus. This analysis also supports the use of the modified, 7-day recall version of the PBC-40 to measure impacts of pruritus among patients with PBC. The use of a questionnaire with a shortened recall period might lead to less recall bias, could be easier for patients to report on, and may be useful in clinical settings.

The Phase 2b GLIMMER trial, the Phase 3 GLISTEN (NCT04950127), and the long-term safety and tolerability (NCT04167358) studies of linerixibat in patients with PBC and pruritus all utilize these validated PRO measures [19, 44, 45]. Using brief, simple PRO measures, such as the NRS items, is important within interventional trials of potential new treatments for PBC-related pruritus to determine treatment-related changes. Furthermore, both the NRS and PBC-40 (7-day recall) can be adapted for use in a clinical care setting. Such use could improve evaluation of PBC-related pruritus and might help healthcare professionals track and assess its severity and impact on their patients over time. Routine use of these tools may also lead to a better understanding of the disease burden of PBC and facilitate patient-centered care and more effective management of the bothersome symptoms associated with PBC.

### Limitations

The observational study had a number of limitations. For example, it recruited participants based on self-reported PBC rather than requiring a physician diagnosis as in GLIMMER; however, this is strengthened by nearly all patients (97%) receiving prescription first-line therapy for the treatment of PBC. Participants also did not need to be experiencing pruritus at the time of study enrollment, so may have been relying on recall of prior experiences when completing the evaluations. However, it is noteworthy that nearly 70% of participants were experiencing pruritus at screening, with only 3% rating their overall pruritus severity as none at the start of the study (Day 1). Including a subset of patients not currently experiencing pruritus in the observational study, alongside those with the targeted symptom (itch) may be an advantage, as it allows for the evaluation of item-level and scale performance of WI-NRS among a group of patients diagnosed with the overall parent condition (PBC), who might approximate the experiences and PRO responses of those for whom pruritus is resolved through treatment. Given the natural variability of the itch symptom, it is also possible that participants who were not experiencing pruritus at the time of enrollment (screening) may have gone on to experience pruritus at some point during the 8-day observational study. Furthermore, participants in the observational study were asked to rate the severity of their itch on Day 1 and this shows that a range of itch severity was experienced by the study sample at study start. Finally, recruitment was conducted through market research, which may have introduced some selection bias.

It should also be noted that the results are not necessarily representative of all patients with PBC and pruritus. Furthermore, there is no established gold standard for objectively measuring pruritus to evaluate the accuracy of WI-NRS in assessing pruritus. However, convergent validity analysis demonstrated that changes in WI-NRS correlated with the observed change in 5-D Itch, a reliable measure of itching which has been validated in patients with chronic pruritus. Lastly, validation of the Worst Itch, Sleep Interference, and Fatigue NRS items and PBC-40 (7-day recall) was carried out in a cohort of patients with PBC and pruritus primarily of moderate-to-severe intensity. Around one-quarter of patients randomized to treatment in GLIMMER had pruritus that did not quite reach moderate intensity according to the WI-NRS at baseline (Week 4), suggesting potential application even to patients with milder pruritus. However, future analyses could explore the validity and utility of the NRS and PBC-40 (7-day recall) measures specifically in patients with mild pruritus in PBC.

## Conclusions

Results from these studies support the reliability, validity, and responsiveness of the NRS items for Worst Itch, pruritus-related Sleep Interference, and Fatigue as well as the modified PBC-40 (7-day recall version) in patients with PBC with moderate-to-severe pruritus. Therefore, the use of these PROs as assessments for patient-centric trial endpoints is appropriate in clinical trials such as the Phase 3 GLISTEN trial to investigate linerixibat for the treatment of pruritus in patients with PBC. Since pruritus in PBC is a symptom experience that is best reported directly by patients, using these tools in an interventional study will enable potential treatment benefits of investigational products to be assessed in a valid and reliable way. Routine use of these validated PRO measures in clinical practice may also enhance patient care in PBC.

## Electronic supplementary material

Below is the link to the electronic supplementary material.

Supplementary Material 1

## Data Availability

Please refer to GSK weblink to access GSK’s data-sharing policies and as applicable seek anonymized subject level data via the link https://www.gsk-studyregister.com/en/.

## Abbreviations

5-D: 5-dimension
ALP: Alkaline phosphatase
ANCOVA: Analysis of covariance
ANOVA: Analysis of variance
BDI-II: Beck Depression Inventory - II
BPI: Brief Pain Inventory
CFI: Comparative fit index
FDA: US Food and Drug Administration
FU: Follow up
HRQoL: Health-related quality of life
ICC: Intraclass correlation coefficients
IQR: Interquartile range
NRS: Numerical rating scale
PBC: Primary biliary cholangitis
PBC-40: Primary Biliary Cholangitis – 40 items
PGI-C: Patient Global Impression of Change
PGI-S: Patient Global Impression of Severity
PRO: Patient-reported outcome
RMSEA: Root mean square error of approximation
SD: Standard deviation
SRM: Standardized response mean
UDCA: Ursodeoxycholic acid
ULN: Upper limit of normal
VAS: Visual analog scale
VRS: Verbal rating scale
WI-NRS: Worst Itch Numerical Rating Scale

## References

[1] European Association for the Study of the Liver. EASL Clinical Practice Guidelines: the diagnosis and management of patients with primary biliary cholangitis. J Hepatol. 2017;67(1):145–72. 10.1016/j.jhep.2017.03.022. 10.1016/j.jhep.2017.03.022 28427765

[2] Hegade VS, Mells GF, Fisher H, Kendrick S, DiBello J, Gilchrist K, Alexander GJ, Hirschfield GM, Sandford RN, Jones DEJ, the UK-PBC Consortium. Pruritus is common and undertreated in patients with primary biliary cholangitis in the united Kingdom. Clin Gastroenterol Hepatol. 2019;17(7):1379–1387.E3. 10.1016/j.cgh.2018.12.007. 30557739 10.1016/j.cgh.2018.12.007

[3] Mayo MJ, Carey E, Smith HT, Mospan AR, McLaughlin M, Thompson A, Morris HL, Sandefur R, Kim WR, Bowlus C, the TARGET-PBC Investigators, Levy C. Impact of pruritus on quality of life and current treatment patterns in patients with primary biliary cholangitis. Dig Dis Sci. 2023;68(3):995–1005. 10.1007/s10620-022-07581-x. 35704252 10.1007/s10620-022-07581-x PMC10406656

[4] Pate J, Gutierrez JA, Frenette CT, Goel A, Kumar S, Manch RA, Mena EA, Pockros PJ, Satapathy SK, Yimam KK, Gish RG. Practical strategies for pruritus management in the obeticholic acid-treated patient with PBC: proceedings from the 2018 expert panel. BMJ Open Gastroenterol. 2019;6(1):e000256. 10.1136/bmjgast-2018-000256 10.1136/bmjgast-2018-000256 PMC6361341 30815273

[5] Oeda S, Takahashi H, Yoshida H, Ogawa Y, Imajo K, Yoneda M, Koshiyama Y, Ono M, Hyogo H, Kawaguchi T, Fujii H, Nishino K, Sumida Y, Tanaka S, Kawanaka M, Torimura T, Saibara T, Kawaguchi A, Nakajima A, Eguchi Y, Japan Study Group of Nonalcoholic Fatty Liver Disease (JSG-NAFLD). Prevalence of pruritus in patients with chronic liver disease: a multicenter study. Hepatol Res. 2018;48(3):E252–62. 10.1111/hepr.12978 28877392 10.1111/hepr.12978

[6] Gungabissoon U, Smith HT, von Maltzahn R, Logie J, Fairburn-Beech J, Ma LPD, McGirr A, Hunnicutt JN, Rowe CL, Tierney M, Friedler HS. Pruritus in primary biliary cholangitis is under-recorded in patient medical records. BMJ Open Gastroenterol. 2024;11(1). 10.1136/bmjgast-2023-001287. 10.1136/bmjgast-2023-001287 PMC10982897 38538090

[7] von Maltzahn R, Mayo MJ, Smith HT, Thompson A, Das S, de Souza AR, Lisi E, Levy C, McLaughlin MM, Jones D. Relationship between pruritus and sleep in participants with primary biliary cholangitis in the phase 2b GLIMMER trial. J Patient Rep Outcomes. 2024;8(1):60. 10.1186/s41687-024-00722-y. 38862718 10.1186/s41687-024-00722-y PMC11166618

[8] Lindor KD, Bowlus CL, Boyer J, Levy C, Mayo M. Primary biliary cholangitis: 2018 practice guidance from the American Association for the Study of Liver Diseases. Hepatology. 2019;69(1):394–419. 10.1002/hep.30145. 30070375 10.1002/hep.30145

[9] Faisal A. Understanding fatigue and pruritus in primary biliary cholangitis. Clin Liver Dis (Hoboken). 2024;23(1):e0216. 10.1097/CLD.0000000000000216. 38831766 10.1097/CLD.0000000000000216 PMC11146472

[10] Gries K, Berry P, Harrington M, Crescioni M, Patel M, Rudell K, Safikhani S, Pease S, Vernon M. Literature review to assemble the evidence for response scales used in patient-reported outcome measures. J Patient Rep Outcomes. 2017;2:41. 10.1186/s41687-018-0056-3. 30238086 10.1186/s41687-018-0056-3 PMC6127075

[11] Verduzco HA, Shirazian S. CKD-Associated pruritus: new insights into diagnosis, pathogenesis, and management. Kidney Int Rep. 2020;5(9):1387–402. 10.1016/j.ekir.2020.04.027. 32954065 10.1016/j.ekir.2020.04.027 PMC7486142

[12] Rams A, Baldasaro J, Bunod L, Delbecque L, Strzok S, Meunier J, ElMaraghy H, Sun L, Pierce E. Assessing itch severity: content validity and psychometric properties of a patient-reported Pruritus Numeric Rating Scale in atopic dermatitis. Adv Ther. 2024;41(4):1512–25. 10.1007/s12325-024-02802-3 38363461 10.1007/s12325-024-02802-3 PMC10960880

[13] Yosipovitch G, Reaney M, Mastey V, Eckert L, Abbe A, Nelson L, Clark M, Williams N, Chen Z, Ardeleanu M, Akinlade B, Graham NMH, Pirozzi G, Staudinger H, Plaum S, Radin A, Gadkari A. Peak Pruritus Numerical Rating Scale: psychometric validation and responder definition for assessing itch in moderate-to-severe atopic dermatitis. Br J Dermatol. 2019;181(4):761–9. 10.1111/bjd.17744. 30729499 10.1111/bjd.17744 PMC6850643

[14] Janmohamed SR, Gwillim EC, Yousaf M, Patel KR, Silverberg JI. The impact of prurigo nodularis on quality of life: a systematic review and meta-analysis. Arch Dermatol Res. 2021;313(8):669–77. 10.1007/s00403-020-02148-0. 33108524 10.1007/s00403-020-02148-0

[15] Erickson S, Kim BS. Research techniques made simple: itch measurement in clinical trials. J Invest Dermatol. 2019;139(2):264–9. 10.1016/j.jid.2018.12.004. e261. 30665580 10.1016/j.jid.2018.12.004 PMC8922716

[16] Food and Drug Administration (FDA). Development of Non-Opioid Analgesics for Acute Pain. Guidance for Industry. 2022. Accessed August 8, 2024. https://www.fda.gov/media/156063/download

[17] Gnanasakthy A, Mordin M, Evans E, Doward L, DeMuro C. A review of patient-reported outcome labeling in the United States (2011–2015). Value Health. 2017;20(3):420–9. 10.1016/j.jval.2016.10.006 28292487 10.1016/j.jval.2016.10.006

[18] Gnanasakthy A, Norcross L, DeMuro Romano C, Carson RT. A review of patient-reported outcome labeling of FDA-approved new drugs (2016–2020): counts, categories, and comprehensibility. Value Health. 2022;25(4):647–55. 10.1016/j.jval.2021.10.006 35365309 10.1016/j.jval.2021.10.006

[19] Levy C, Kendrick S, Bowlus CL, Tanaka A, Jones D, Kremer AE, Mayo MJ, Haque N, von Maltzahn R, Allinder M, Swift B, McLaughlin MM, Hirschfield GM, GLIMMER Study Group. GLIMMER: a randomized phase 2b dose-ranging trial of linerixibat in primary biliary cholangitis patients with pruritus. Clin Gastroenterol Hepatol. 2023;21(7):1902–1912.E13. 10.1016/j.cgh.2022.10.032 10.1016/j.cgh.2022.10.032 36343847

[20] Bowlus CL, Eksteen B, Cheung AC, Thorburn D, Moylan CA, Pockros PJ, Forman LM, Dorenbaum A, Hirschfield GM, Kennedy C, Jaecklin T, McKibben A, Chien E, Baek M, Vig P, Levy C. Safety, tolerability, and efficacy of maralixibat in adults with primary sclerosing cholangitis: open-label pilot study. Hepatol Commun. 2023;7(6):e0153. 10.1097/HC9.0000000000000153 10.1097/HC9.0000000000000153 PMC10187837 37184523

[21] Foster B, Andrae D, Vig P, McKibben A, Bowlus C. Psychometric evaluation of the Adult Itch Reported Outcome tool, a worst itch numeric rating scale in adults with cholestatic liver disease. The Digital International Liver Congress™ (Poster no. FRI131). 2020. https://mirumpharma.com/wp-content/uploads/2021/06/b6ed837977fb-Adult_ItchRO_poster_EASL_2020_FINAL_14Jul20_SENT_TO_EOs.pdf

[22] Gordon SC, Trudeau S, Regev A, Uhas JM, Chakladar S, Pinto-Correia A, Gottlieb K, Schlichting D. Baricitinib and primary biliary cholangitis. J Transl Autoimmun. 2021;4:100107. 10.1016/j.jtauto.2021.100107. 34195587 10.1016/j.jtauto.2021.100107 PMC8240017

[23] Hegade VS, Kendrick SF, Dobbins RL, Miller SR, Thompson D, Richards D, Storey J, Dukes GE, Corrigan M, Oude Elferink RP, Beuers U, Hirschfield GM, Jones DE. Effect of ileal bile acid transporter inhibitor GSK2330672 on pruritus in primary biliary cholangitis: a double-blind, randomised, placebo-controlled, crossover, phase 2a study. Lancet. 2017;389(10074):1114–23. 10.1016/S0140-6736(17)30319-7. 28187915 10.1016/S0140-6736(17)30319-7

[24] Hirschfield GM, Bowlus CL, Mayo MJ, Kremer AE, Vierling JM, Kowdley KV, Levy C, Villamil A, Ladron de Guevara Cetina AL, Janczewska E, Zigmond E, Jeong SH, Yilmaz Y, Kallis Y, Corpechot C, Buggisch P, Invernizzi P, Londono Hurtado MC, Bergheanu S, Yang K, Choi YJ, Crittenden DB, McWherter CA, RESPONSE Study Group. A phase 3 trial of seladelpar in primary biliary cholangitis. N Engl J Med. 2024;390(9):783–94. 10.1056/NEJMoa2312100 38381664 10.1056/NEJMoa2312100

[25] Hirschfield GM, Shiffman ML, Gulamhusein A, Kowdley KV, Vierling JM, Levy C, Kremer AE, Zigmond E, Andreone P, Gordon SC, Bowlus CL, Lawitz EJ, Aspinall RJ, Pratt DS, Raikhelson K, Gonzalez-Huezo MS, Heneghan MA, Jeong SH, Ladrón de Guevara AL, Mayo MJ, Dalekos GN, Drenth JPH, Janczewska E, Leggett BA, Nevens F, Vargas V, Zuckerman E, Corpechot C, Fassio E, Hinrichsen H, Invernizzi P, Trivedi PJ, Forman L, Jones DEJ, Ryder SD, Swain MG, Steinberg A, Boudes PF, Choi YJ, McWherter CA. Seladelpar efficacy and safety at 3 months in patients with primary biliary cholangitis: ENHANCE, a phase 3, randomized, placebo-controlled study. Hepatology. 2023;78(2):397–415. 10.1097/hep.0000000000000395. 37386786 10.1097/HEP.0000000000000395 PMC10344437

[26] Kowdley KV, Bowlus CL, Levy C, Akarca US, Alvares-da-Silva MR, Andreone P, Arrese M, Corpechot C, Francque SM, Heneghan MA, Invernizzi P, Jones D, Kruger FC, Lawitz E, Mayo MJ, Shiffman ML, Swain MG, Valera JM, Vargas V, Vierling JM, Villamil A, Addy C, Dietrich J, Germain JM, Mazain S, Rafailovic D, Tadde B, Miller B, Shu J, Zein CO, Schattenberg JM, ELATIVE Study Investigators’ Group. Efficacy and safety of elafibranor in primary biliary cholangitis. N Engl J Med. 2024;390(9):795–805. 10.1056/NEJMoa2306185. 37962077 10.1056/NEJMoa2306185

[27] Mayo MJ, Pockros PJ, Jones D, Bowlus CL, Levy C, Patanwala I, Bacon B, Luketic V, Vuppalanchi R, Medendorp S, Dorenbaum A, Kennedy C, Novak P, Gu J, Apostol G, Hirschfield GM. A randomized, controlled, phase 2 study of maralixibat in the treatment of itching associated with primary biliary cholangitis. Hepatol Commun. 2019;3(3):365–81. 10.1002/hep4.1305. 30859149 10.1002/hep4.1305 PMC6396374

[28] Atkinson TM, Mendoza TR, Sit L, Passik S, Scher HI, Cleeland C, Basch E. The brief pain inventory and its “pain at its worst in the last 24 hours” item: clinical trial endpoint considerations. Pain Med. 2010;11(3):337–46. 10.1111/j.1526-4637.2009.00774.x. 20030743 10.1111/j.1526-4637.2009.00774.x PMC3806650

[29] Jacoby A, Rannard A, Buck D, Bhala N, Newton JL, James OF, Jones DE. Development, validation, and evaluation of the PBC-40, a disease specific health related quality of life measure for primary biliary cirrhosis. Gut. 2005;54(11):1622–9. 10.1136/gut.2005.065862. 15961522 10.1136/gut.2005.065862 PMC1774759

[30] Martin ML, Stassek L, Blum SI, Joshi AV, Jones D. Development and adaptation of patient-reported outcome measures for patients who experience itch associated with primary biliary cholangitis. J Patient Rep Outcomes. 2019;3(1):2. 10.1186/s41687-018-0090-1. 30645706 10.1186/s41687-018-0090-1 PMC6333594

[31] de Veer RC, da Silva G, van Hooff MC, Harms MH, Metselaar HJ, Willemse J, Utomo E, van der Meer AJ. Measurement properties of the PBC-40 and PBC-27: a Dutch validation study. BMJ Open Gastroenterol. 2021;8(1):e000758. 10.1136/bmjgast-2021-000758. 10.1136/bmjgast-2021-000758 PMC8679126 34916226

[32] Jones D, Carbone M, Invernizzi P, Little N, Nevens F, Swain MG, Wiesel P, Levy C. Impact of setanaxib on quality of life outcomes in primary biliary cholangitis in a phase 2 randomized controlled trial. Hepatol Commun. 2023;7(3):e0057. 10.1097/HC9.0000000000000057. 36809195 10.1097/HC9.0000000000000057 PMC9949832

[33] Liu YS, Jia G, Ding DW, Zheng LH, Sun RQ, Wang XF, Deng J, Yang CM, Cui LN, Guo CC, Shang YL, Han Y. Differences in the perceptions of patients with primary biliary cholangitis and physicians from various hospital departments: an online survey. J Dig Dis. 2024;25(1):61–9. 10.1111/1751-2980.13254. 38408848 10.1111/1751-2980.13254

[34] Elman S, Hynan LS, Gabriel V, Mayo MJ. The 5-D itch scale: a new measure of pruritus. Br J Dermatol. 2010;162(3):587–93. 10.1111/j.1365-2133.2009.09586.x. 19995367 10.1111/j.1365-2133.2009.09586.x PMC2875190

[35] Rabin R, de Charro F. EQ-5D: a measure of health status from the EuroQol group. Ann Med. 2001;33(5):337–43. 10.3109/07853890109002087. 11491192 10.3109/07853890109002087

[36] Hays R, Revicki DA. Reliability and validity (including responsiveness). New York: Oxford University Press; 2005.

[37] Yu C. (2002) Evaluating cutoff criteria of model fit indices for latent variable models with binary and continuous outcomes [Doctoral Dissertation]. University of California, Los Angeles.

[38] Steiger J. Understanding the limitations of global fit assessment in structural equation modeling. Pers Individ Dif. 2007;42(5):893–8. 10.1016/j.paid.2006.09.017.

[39] Hooper D, Coughlan J, Mullen M. Structural equation modelling: guidelines for determining model fit. Electron J Bus Res Methods. 2008;6(1):53–60.

[40] Puelles J, Fofana F, Rodriguez D, Silverberg JI, Wollenberg A, Dias Barbosa C, Vernon M, Chavda R, Gabriel S, Piketty C. Psychometric validation and responder definition of the sleep disturbance numerical rating scale in moderate-to-severe atopic dermatitis. Br J Dermatol. 2022;186(2):285–94. 10.1111/bjd.20783. 34608623 10.1111/bjd.20783 PMC9299666

[41] Silverberg JI, DeLozier A, Sun L, Thyssen JP, Kim B, Yosipovitch G, Nunes FP, Gugiu PC, Doll HA, Eichenfield LF. Psychometric properties of the itch numeric rating scale, skin pain numeric rating scale, and atopic dermatitis sleep scale in adult patients with moderate-to-severe atopic dermatitis. Health Qual Life Outcomes. 2021;19(1):247. 10.1186/s12955-021-01877-8. 34688290 10.1186/s12955-021-01877-8 PMC8542315

[42] Kimel M, Zeidler C, Kwon P, Revicki D, Stander S. Validation of psychometric properties of the itch numeric rating scale for pruritus associated with prurigo nodularis: a secondary analysis of a randomized clinical trial. JAMA Dermatol. 2020;156(12):1354–8. 10.1001/jamadermatol.2020.3071 32936233 10.1001/jamadermatol.2020.3071 PMC7495327

[43] Vernon MK, Swett LL, Speck RM, Munera C, Spencer RH, Wen W, Menzaghi F. Psychometric validation and meaningful change thresholds of the Worst Itching Intensity Numerical Rating Scale for assessing itch in patients with chronic kidney disease-associated pruritus. J Patient Rep Outcomes. 2021;5(1):134. 10.1186/s41687-021-00404-z. 34952964 10.1186/s41687-021-00404-z PMC8709801

[44] ClinicalTrials.gov. Global Linerixibat Itch Study of Efficacy and Safety in Primary Biliary Cholangitis (PBC) (GLISTEN). Identifier NCT04950127. https://clinicaltrials.gov/study/NCT04950127?term=NCT04950127&rank=1 Accessed March 12, 2025.

[45] ClinicalTrials.gov. Linerixibat Long-term Safety, and Tolerability Study (LLSAT). Identifier NCT04167358. https://clinicaltrials.gov/study/NCT04167358?term=NCT04167358&rank=1 Accessed October 17, 2024.

