# Supplementary material for: Psychometric validation of the Worst Itch Numerical Rating Scale (WI-NRS) and other patient-reported outcome measures for assessing severity and impact of pruritus in patients with primary biliary cholangitis
Source: Orphanet J Rare Dis. 2025 Jul 31;20:390. doi: 10.1186/s13023-025-03798-x (PMC12315290; doi:10.1186/s13023-025-03798-x)
Supplement: Supplementary file 1 — Supplementary Material 1 [file 13023_2025_3798_MOESM1_ESM.docx]

# Supplementary Figures

## Supplementary Fig. 1. Known-groups validity of PBC-40 (7-day recall) at Week 16 in GLIMMER for (A) tiredness interference with daily activities^a^ and (B) EQ-5D VAS


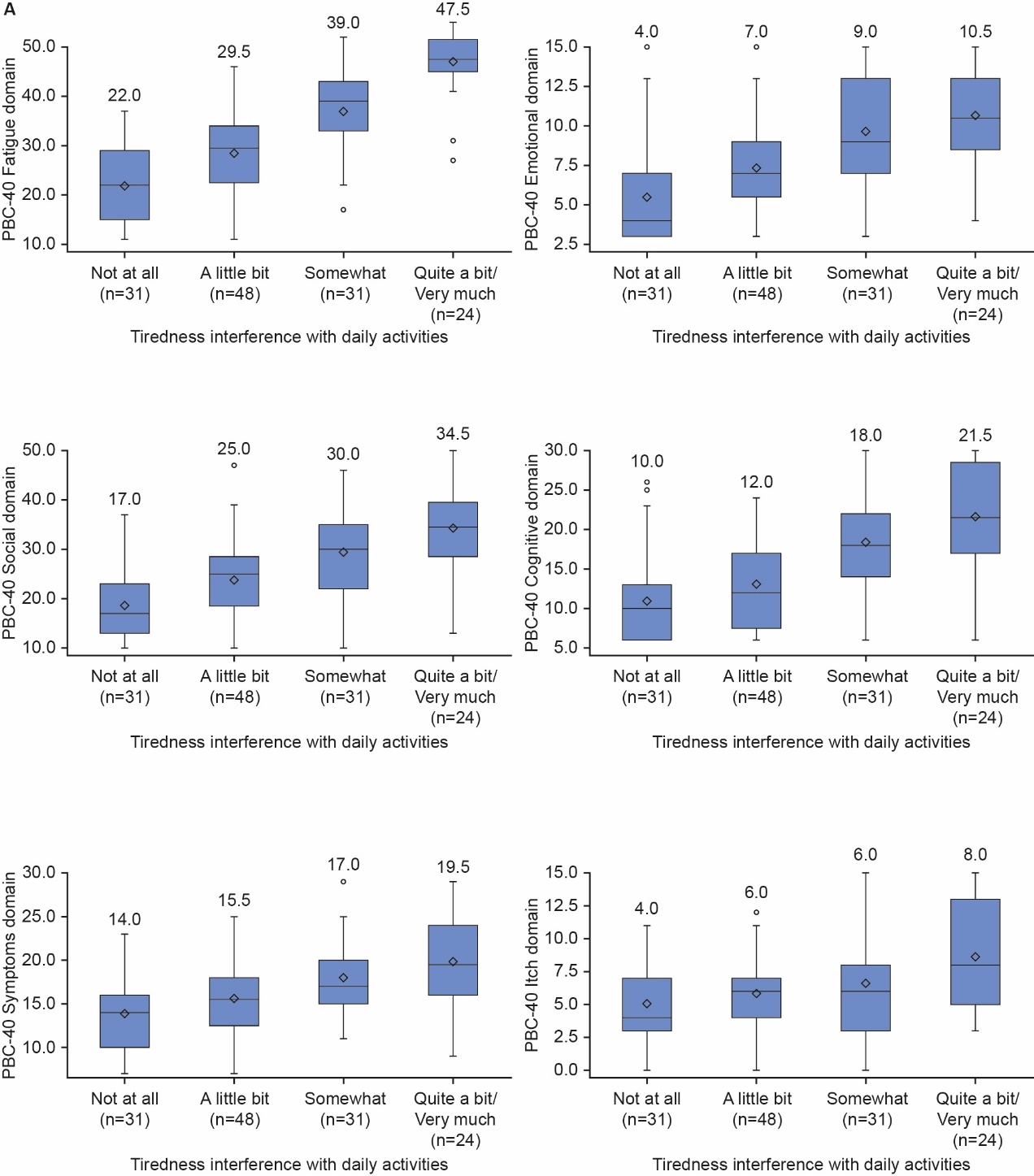


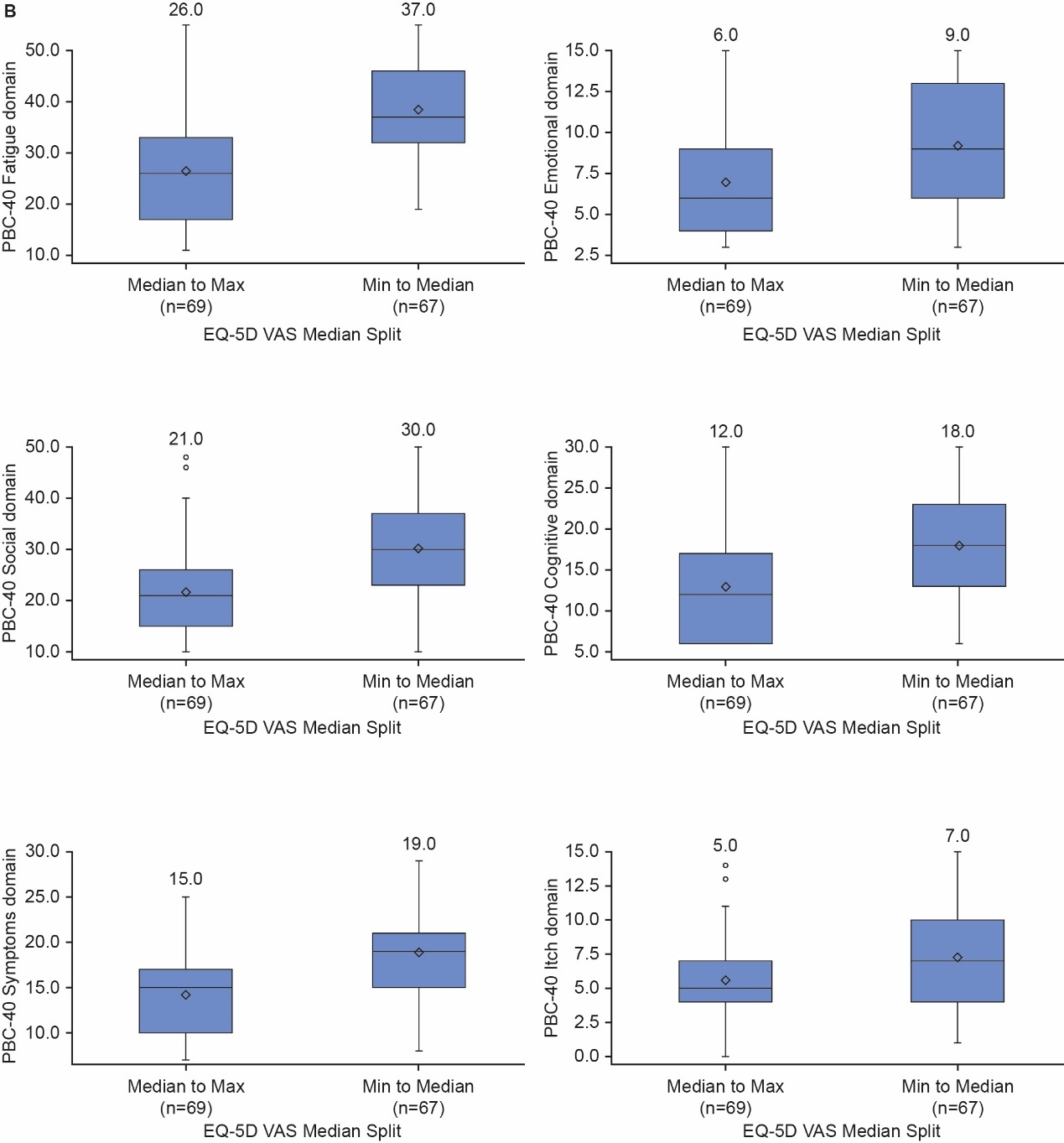


The numbers above the box plots represent medians.

^a^Question asked via an eDiary: How much did tiredness interfere with your daily activities today? (Not at all, A little, Somewhat, Quite, Extremely).
PBC-40, Primary Biliary Cholangitis – 40 Items; VAS, visual analog scale.

# Supplementary Tables

## Supplementary Table 1. GLIMMER PRO instruments and assessment schedule

|  |  | | **Screening** | **Initial study period** | **Main study period** | | | | **Final study period** | **FU period** | **Early End of**  **Treatment or**  **Study**  **Withdrawal**  **Assessments^a^** |
| --- | --- | --- | --- | --- | --- | --- | --- | --- | --- | --- | --- |
|  | **Visit** | | **Visit 1 (Screening)** | **Visit 2 (Day 1)** | **Visit 3 (baseline/ Week 4)** | **Visit 4 (Week 8)** | **Visit 5 (Week 12)** | **Visit 6 (Week 16)** | **Visit 7 (Week 20)** | **Visit 8 (end of FU phone visit)** |  |
|  | **Day number (window)** | | **Day -45 to -1** | **Day 1** | **Day 28 (28 to 35)** | **Day 56 (56 to 63)** | **Day 84 (84 to 91)** | **Day 112 (112 to 119)** | **Day 140 (140 to 147)** | **Day 168 (168 to 175)** |  |
| **PRO** | **Description** | **Response scale(s)** |  | | | | | | | | |
| **Symptom questionnaire eDiary^b^** | An 18-item, self-reported questionnaire, administered in the form of a daily diary (completed morning and evening), designed to record participants’ symptoms of itch and its impact on sleep and level of fatigue Key NRS items included: Rate worst itching (WI-NRS)  Rate how itching interfered with sleep (Sleep Interference NRS)  Rate worst level of fatigue (Fatigue NRS) | Itch, Sleep Interference and Fatigue NRS scored on 0–10 NRS |  | X–––––––––––––––––––––––––––––––––––––––––––––––––––––––X | | | | | |  |  |
| **PBC-40** | A 40-item, self-reported, validated disease-specific scale designed to assess HRQoL in participants with PBC (7-day recall) | Domains of Fatigue, Emotional, Social, Cognitive, Symptoms, and Itch scored on a 5-point Likert scale | X | X | X | X | X | X | X |  | X |
| **5-D Itch scale** | A brief, self-reported, multidimensional questionnaire designed to measure itch and its impact on daily life using a 2-week recall period | Domains of duration, degree, direction, and disability measured on 5-point Likert scale,^c^ plus domain of distribution covering 16 body regions |  | X | X |  |  | X | X |  | X |
| **EQ-5D-5L** | A self-reported, generic, utility-scale developed by the EuroQoL Group and designed to measure health states | Mobility, self-care, usual activities, pain/discomfort, and anxiety/depression scored on 5-point Likert scale, plus VAS to assess health status |  | X | X |  |  | X |  |  | X |
| **PGI-S** | A single-item, self-reported, generic scale to assess the subject’s impression of itch  severity | Measures itch severity on a 5-point Likert scale |  | X | X | X | X | X | X |  | X |
| **PGI-C** | A self-reported, generic scale to assess the subject’s impression of change in itch since the start of the study. A follow-up question asked participants whether this change was meaningful or not (Yes/No) | Measures change in itch on a 7-point Likert scale |  |  | X | X | X | X | X |  | X |
| **BDI-II** | A 21-item, self-reported scale designed to measure the severity of depression in adults and adolescents during the past 2 weeks including today | Cognitive-affective and somatic items scored on a 4-point Likert scale | X |  | X |  |  | X |  |  | X |

^a^Early End of Treatment Assessments were completed for participants who prematurely discontinued study treatment following randomization. Study Withdrawal Assessments were completed for participants who withdraw consent for any further participation in the study following randomization. Note: If a participant discontinued study treatment at the same time they withdrew from the study, only the End of Treatment Assessments was required
^b^Symptom questionnaire included the NRS items
^c^3 items on disability scale are measured on 6-point Likert scale

5-D, 5-dimension; BDI-II, Beck Depression Inventory-II; FU, follow-up; HRQoL, health-related quality of life; NRS, numerical rating scale; PGI-C, Patient Global Impression of Change; PGI-S, Patient Global Impression of Severity; PBC, primary biliary cholangitis; PBC-40, Primary Biliary Cholangitis – 40 Items; PRO, patient-reported outcome; VAS, visual analog scale; WI-NRS, Worst Itch NRS.

## Supplementary Table 2. PRO instruments and assessment schedule in the observational study

| **Data Collection Measure** | **Description** | **Response scale(s)** | **Day 1** | **Day 2** | **Day 3** | **Day 4** | **Day 5** | **Day 6** | **Day 7** | **Day 8** |
| --- | --- | --- | --- | --- | --- | --- | --- | --- | --- | --- |
| **Symptom Questionnaire^a^** | An 18-item, self-reported questionnaire, administered in the form of a daily diary (completed morning and evening), designed to record participants’ symptoms of itch and its impact on sleep and level of fatigue  Key NRS items included: Rate worst itching (WI-NRS)  Rate how itching interfered with sleep (Sleep Interference NRS)  Rate worst level of fatigue (Fatigue NRS) | Itch, sleep and fatigue scored on 0–10 NRS | X | X | X | X | X | X | X | X |
| **PBC-40** | A 40-item, self-reported, validated disease-specific scale designed to assess HRQoL in participants with PBC (7-day recall) | Domains of Fatigue, Emotional, Social, Cognitive, Symptoms, and Itch scored on a 5-point Likert scale | X |  |  |  |  |  |  | X |
| **PGI-S** | A single-item, self-reported, generic scale to assess the subject’s impression of itch severity | Measures itch severity on a 5-point Likert scale | X |  |  |  |  |  |  | X |
| **5-D Itch scale** | A brief, self-reported, multidimensional questionnaire designed to measure itch and its impact on daily life using a 2-week recall period | Domains of duration, degree, direction, and disability measured on 5-point Likert scale (with 3 items on disability scale measured on 6-point Likert scale), plus domain of distribution covering 16 body regions | X |  |  |  |  |  |  | X |
| **PROMIS-43** | A 43-item, self-reported questionnaire covering seven health HRQoL domains | Domains of depression, anxiety, physical function, pain interference, fatigue, sleep disturbance, and ability to participate in social roles and activities measured on 5-point scale | X |  |  |  |  |  |  | X |
| **PGI-C** | A self-reported, generic scale to assess the subject’s impression of change in itch since the start of the study. A follow-up question asked participants whether this change was meaningful or not (Yes/No) | Measures change on a 7-point Likert scale |  |  |  |  |  |  |  | X |
| **EQ-5D-3L** | A self-reported, generic, utility-scale developed by the EuroQoL Group and designed to measure health states | Mobility, self-care, usual activities, pain/discomfort, and anxiety/depression scored on 3-point scale, plus VAS to assess health status | X |  |  |  |  |  |  | X |

^a^Questionnaire completed morning and evening, including the NRS items

5-D, 5-dimension; HRQoL, health-related quality of life; NRS, numerical rating scale; PBC, primary biliary cholangitis; PBC-40, Primary Biliary Cholangitis – 40 Items; PGI-C, Patient Global Impression of Change; PGI-S, Patient Global Impression of Severity; PRO, patient-reported outcome; PROMIS®, Patient Reported Outcomes Measurement Information System; VAS, visual analog scale; WI-NRS, Worst Itch NRS.

## Supplementary Table 3. Confirmatory factor analysis of the PBC-40 (7-day recall) at Day 1 in the observational study

| **PBC-40 (7-day recall) domain** | **Items** | **Factor coefficient range** | **RMSEA**  **(90% CI)** | **CFI** |
| --- | --- | --- | --- | --- |
| Fatigue | 11 | 0.72–0.94 | 0.059 (0.051–0.066) | 0.966 |
| Emotional | 3 | 0.56–0.87 |  |  |
| Social | 10 | 0.43–0.91 |  |  |
| Cognitive | 6 | 0.87–0.92 |  |  |
| Symptoms | 7 | 0.39–0.76 |  |  |
| Itch | 3 | 0.82–0.92 |  |  |

CFI, comparative fit index; CI, confidence interval; PBC-40, Primary Biliary Cholangitis – 40 items;
RMSEA, root mean square error of approximation.

## Supplementary Table 4. Internal consistency reliability of PBC-40 (7-day recall) at baseline in GLIMMER and at Day 1 in the observational study

| **PBC-40 (7-day recall) domains** | **GLIMMER baseline**  **Cronbach’s alpha  (N=147)** | **Observational study Day 1  Cronbach’s alpha (N=141)** |
| --- | --- | --- |
| Overall alpha | - | 0.95 |
| Fatigue | 0.96 | 0.95 |
| Emotional | 0.82 | 0.78 |
| Social | 0.88 | 0.87 |
| Cognitive | 0.97 | 0.95 |
| Symptoms | 0.67 | 0.73 |
| Itch | 0.76 | 0.87 |

Cronbach’s alpha was calculated for raw variables

PBC-40, Primary Biliary Cholangitis – 40 items.

## Supplementary Table 5. Test–retest reliability of WI-NRS, Sleep Interference NRS and Fatigue NRS in stable participants in GLIMMER

|  | **N** | **Time 1^a^ Mean (SD)** | **Time 2^b^ Mean (SD)** | **Mean difference^c^** | **t-value of paired t-test** | **p-value^d^** | **ICC**  **(Time 1 – Time 2)** |
| --- | --- | --- | --- | --- | --- | --- | --- |
| WI-NRS  (weekly itch score) | 145 | 5.8 (1.9) | 5.5 (2.0) | −0.3 | −2.88 | 0.0046 | 0.81 |
| Sleep Interference NRS  (weekly sleep score) | 145 | 4.2 (2.5) | 3.9 (2.5) | −0.2 | −2.17 | 0.0314 | 0.85 |
| Fatigue NRS  (weekly fatigue score) | 145 | 5.3 (2.3) | 5.2 (2.5) | −0.1 | −1.24 | 0.2153 | 0.88 |

Stable participants were defined as those who selected the same PGI-S response at Time 1 and Time 2. Test–retest reliability was conducted using data from the initial study period when patients were not on the active study treatment as the least amount of change was anticipated during this timeframe.

^a^Time 1 = Days 2–8 (WI-NRS); Day 2 (Sleep Interference NRS; Fatigue NRS)
^b^Time 2 = Days 9–15 (WI-NRS); 1 week later (Sleep Interference NRS and Fatigue NRS)
^c^Time 2 – Time 1

^d^p-value for paired t-test

ICC, intraclass correlation coefficient; NRS, numerical rating scale; PGI-S, Patient Global Impression of Severity; SD, standard deviation; WI-NRS, Worst Itch NRS.

## Supplementary Table 6. Additional test–retest reliability of WI-NRS in stable participants in GLIMMER and test–retest reliability of WI-NRS, Sleep Interference NRS and Fatigue NRS in stable participants in the observational study

|  | **N** | **Time 1^a^ Mean (SD)** | **Time 2^b^ Mean (SD)** | **Mean difference^c^** | **t-value of paired t-test** | **p-value^d^** | **ICC**  **(Time 1 – Time 2)** |
| --- | --- | --- | --- | --- | --- | --- | --- |
| **GLIMMER** | | | | | | | |
| WI-NRS  (weekly itch score) | 68 | 5.5 (2.0) | 5.5 (2.0) | −0.1 | −0.49 | 0.6260 | 0.73 |
| **Observational study** | | | | | | | |
| WI-NRS  (daily itch score) | 78 | 3.5 (2.5) | 3.3 (2.6) | −0.2 | −1.16 | 0.2510 | 0.78 |
| Sleep Interference NRS  (daily sleep score) | 77 | 2.6 (2.7) | 2.2 (2.5) | −0.5 | −2.35 | 0.0212 | 0.77 |
| Fatigue NRS  (daily fatigue score) | 77 | 5.2 (2.6) | 4.8 (2.7) | −0.3 | −1.44 | 0.1549 | 0.72 |

Stable participants were defined as those who selected the same PGI-S response at Time 1 and Time 2.

^a^Time 1 = Day 2 (GLIMMER); Day 1 (observational study)
^b^Time 2 = Baseline/Week 4 (GLIMMER); Day 8 (observational study) ^c^Time 2 – Time 1

^d^p-value for paired t-test

ICC, intraclass correlation coefficient; NRS, Numerical Rating Scale; PGI-S, Patient Global Impression of Severity; SD, standard deviation.

## Supplementary Table 7. Test–retest reliability of PBC-40 (7-day recall) in stable participants in GLIMMER and the observational study

| **PBC-40 domain** | **N** | **Time 1^a^**  **Mean (SD)** | **Time 2^b^**  **Mean (SD)** | **Mean difference^c^** | **t-value of paired t-test** | **p-value^d^** | **ICC** |
| --- | --- | --- | --- | --- | --- | --- | --- |
|  | **GLIMMER** | | | | | | |
| Fatigue | 144 | 33.6 (11.7) | 33.8 (10.6) | 0.2 | 0.44 | 0.6571 | 0.87 |
| Emotional | 144 | 9.5 (3.6) | 9.1 (3.6) | −0.4 | −2.01 | 0.0464 | 0.72 |
| Social | 144 | 27.1 (9.4) | 27.3 (9.1) | 0.2 | 0.38 | 0.7025 | 0.84 |
| Cognitive | 144 | 15.4 (7.1) | 15.5 (6.5) | 0.1 | 0.45 | 0.6567 | 0.86 |
| Symptoms | 144 | 17.9 (5.3) | 17.6 (4.8) | −0.2 | −0.76 | 0.4493 | 0.77 |
| Itch | 144 | 10.0 (3.3) | 9.7 (3.1) | −0.4 | −2.12 | 0.0354 | 0.79 |
|  | **Observational study** | | | | | | |
| Fatigue | 78 | 34.5 (10.3) | 34.4 (10.2) | −0.1 | −0.15 | 0.8818 | 0.90 |
| Emotional | 78 | 9.8 (2.8) | 9.8 (2.9) | 0.0 | 0.22 | 0.8298 | 0.85 |
| Social | 78 | 29.8 (9.4) | 30.6 (9.0) | 0.8 | 1.40 | 0.1656 | 0.85 |
| Cognitive | 78 | 17.1 (6.4) | 16.5 (5.8) | −0.6 | −1.79 | 0.0781 | 0.89 |
| Symptoms | 78 | 17.4 (4.5) | 17.1 (4.7) | −0.3 | −1.29 | 0.2022 | 0.88 |
| Itch | 78 | 7.0 (3.4) | 6.8 (3.0) | −0.2 | −0.96 | 0.3412 | 0.85 |

Stable subjects defined as those who selected the same PGI-S response at Time 1 and Time 2.

^a^Time 1 = Day -21 to -7 (GLIMMER); Day 1 (observational study)
^b^Time 2 = Day 1 (GLIMMER); Day 8 (observational study)
^c^Mean difference = Time 2 – Time 1

^d^p-value for paired t-test

ICC, intraclass correlation coefficient; PBC-40, Primary Biliary Cholangitis – 40 items; PGI-S, Patient Global Impression of Severity; SD, standard deviation.

## Supplementary Table 8. Convergent validity of PBC-40 (7-day recall) at baseline in GLIMMER and at Day 1 in the observational study

| **PBC-40 (7-day recall) domains^a^** | **r^b^** |
| --- | --- |
| **Fatigue** | |
| Fatigue NRS (GLIMMER)^c^ | 0.69**** |
| Fatigue NRS (observational study) | 0.68**** |
| Sleep Interference NRS (observational study) | 0.27** |
| **Emotional** | |
| BDI-II – total score (GLIMMER) | 0.74**** |
| PROMIS-43 (Depression, Anxiety)  (observational study) | 0.61**** |
| **Social** | |
| PROMIS-43 (Social roles)  (observational study) | \|0.80\|**** |
| **Cognitive** | |
| Concentration item (GLIMMER)^d^ | 0.68**** |
| Concentration item (observational study)^d^ | 0.64**** |
| Remember item (GLIMMER)^e^ | 0.76**** |
| Remember item (observational study)^e^ | 0.69**** |
| **Itch** | |
| 5-D Itch scale total score (GLIMMER) | 0.67**** |
| 5-D Itch scale total score (observational study) | 0.70**** |
| WI-NRS (GLIMMER)^f^ | 0.50**** |
| WI-NRS (observational study)^g^ | 0.79**** |

Negative r values are represented as |value|.
^a^The Symptoms domain was deliberately excluded from evaluation due to the absence of appropriate measures for assessment
^b^Significance levels of Spearman rank-order correlations, p-values are: **<0.01, ****p<0.0001
^c^Weekly fatigue score ^d^How difficult was it for you to concentrate today? (Not at all, A little, Somewhat, Quite, Extremely)
^e^How difficult was it for you to remember things today? (Not at all, A little, Somewhat, Quite, Extremely)
^f^Weekly itch score.

^g^Worst daily itch score
5-D, 5-dimension; BDI-II, Beck Depression Inventory - II; NRS, numerical rating Scale; PBC-40, Primary Biliary Cholangitis – 40 items; PROMIS®, Patient Reported Outcomes Measurement Information System; WI‑NRS, Worst Itch NRS.

## Supplementary Table 9. Known-groups validity of the NRS items at Day 1 in the observational study

|  | **PGI-S** | | | | | |
| --- | --- | --- | --- | --- | --- | --- |
| **PRO (mean score [SD])** | **Absent (n=19)** | **Mild (n=70)** | **Moderate (n=40)** | **Severe (n=12)** | **F Value and significance^a^** | **Post hoc comparison^b^** |
| WI-NRS^c^ | 0.4 (0.8) | 3.3 (1.8) | 5.8 (1.4) | 8.0 (1.1) | 81.6*** | 1***, 2***, 3***, 4***, 5***, 6*** |
| Sleep Interference NRS^d^ | 0.0 (0.0) | 1.9 (2.0) | 5.0 (2.1) | 7.8 (1.2) | 70.8*** | 1**, 2***, 3***, 4***, 5***, 6*** |
| Fatigue NRS^e^ | 3.3 (2.4) | 5.5 (2.5) | 6.5 (1.7) | 6.8 (2.6) | 8.9*** | 1**, 2***, 3** |

The PGI-S has a “very severe” category; however, no one selected that category.
^a^An ANOVA
^b^One-way ANOVA using Scheffe’s Method. 1 = Absent vs mild; 2 = Absent vs moderate; 3 = Absent vs severe; 4 = Mild vs moderate; 5 = Mild vs severe;
6 = Moderate vs severe
^c^Worst daily itch score
^d^Daily sleep score
^e^Daily fatigue score.
P-values are: **<0.01, ***<0.001.
ANOVA, analysis of variance; NRS, numerical rating scale; PGI-S, Patient Global Impression of Severity; PRO, patient-reported outcome; SD, standard deviation; WI-NRS, Worst Itch NRS.

## Supplementary Table 10. Additional responsiveness of PBC-40 (7-day recall) Itch domain from baseline to Week 16 in GLIMMER

|  | **N** | **Baseline Mean (SD)** | **Week 16 Mean (SD)** | **Change**  **Mean (SD)**  **(Week 16 – baseline)** | **Effect size^a^** | **SRM^b^** | **Paired t-test (p-value)^c^** | **F Value  (p-value)^d^** |
| --- | --- | --- | --- | --- | --- | --- | --- | --- |
| PGI-S – Change category^e^ |  |  |  |  |  |  |  | 24.93 (<0.0001) |
| Improved | 83 | 9.5 (3.2) | 5.7 (3.2) | −3.8 (3.1) | −1.2 | −1.2 | −11.4 (<0.0001) |  |
| No change | 42 | 8.2 (3.2) | 7.4 (3.4) | −0.8 (2.5) | −0.2 | −0.3 | −1.99 (0.0529) |  |
| Worsened | 11 | 8.2 (2.6) | 7.9 (3.3) | −0.3 (1.9) | −0.1 | −0.1 | −0.48 (0.6446) |  |

^a^Mean difference between the baseline and Week 16 score divided by SD of the baseline PBC-40 domain scores
^b^Mean difference between the baseline and Week 16 score divided by SD of the change from baseline in PBC-40 domain score
^c^Paired t-test is for the differences in means between the baseline and the follow-up time point
^d^ANCOVA adjusted for baseline PBC-40 domain score was used

^e^“Improved” (score change<0), “unchanged” (score change=0), “worsened” (score change>0)
ANCOVA, analysis of covariance; PBC-40, Primary Biliary Cholangitis − 40 Items; PGI-S, Patient Global Impression of Severity; SD, standard deviation; SRM, standardized response mean.
